# Oxidative Stress and Neonatal Respiratory Extracorporeal Membrane Oxygenation

**DOI:** 10.3389/fphys.2018.01739

**Published:** 2018-12-04

**Authors:** Genny Raffaeli, Stefano Ghirardello, Sofia Passera, Fabio Mosca, Giacomo Cavallaro

**Affiliations:** ^1^NICU, Fondazione IRCCS Ca’ Granda Ospedale Maggiore Policlinico, Milan, Italy; ^2^Department of Clinical Sciences and Community Health, Università degli Studi di Milano, Milan, Italy

**Keywords:** antioxidants, cardiopulmonary bypass, CRRT, ECMO, extracorporeal circulation, oxidative stress

## Abstract

Oxidative stress is a frequent condition in critically ill patients, especially if exposed to extracorporeal circulation, and it is associated with worse outcomes and increased mortality. The inflammation triggered by the contact of blood with a non-endogenous surface, the use of high volumes of packed red blood cells and platelets transfusion, the risk of hyperoxia and the impairment of antioxidation systems contribute to the increase of reactive oxygen species and the imbalance of the redox system. This is responsible for the increased production of superoxide anion, hydrogen peroxide, hydroxyl radicals, and peroxynitrite resulting in increased lipid peroxidation, protein oxidation, and DNA damage. The understanding of the pathophysiologic mechanisms leading to redox imbalance would pave the way for the future development of preventive approaches. This review provides an overview of the clinical impact of the oxidative stress during neonatal extracorporeal support and concludes with a brief perspective on the current antioxidant strategies, with the aim to focus on the potential oxidative stress-mediated cell damage that has been implicated in both short and long-term outcomes.

## Introduction

Extracorporeal membrane oxygenation is life support for reversible heart and/or lung failure refractory to standard treatment, and it is used as bridge-to-recovery, bridge-to-bridge, bridge-to-transplant, or bridge-to-decision (Brogan et al., 2017). This technique was developed by the CPB technology, and it is based on a partial/full bypass of the native cardiac and/or pulmonary function. Neonatal respiratory ECMO was introduced by Robert Bartlett during the 1970s for severe hypoxemic respiratory failure secondary to MAS, PPH and RDS (Bartlett et al., 1976). Despite the progress in neonatal care following the introduction of new therapies and ventilatory strategies, ECMO remains life-saving support for about 600–800 neonates each year worldwide (Thiagarajan et al., 2017). Across the world, from 1989 to 2017, about 27.238 newborns needed an ECMO procedure for respiratory failure, 7.592 for cardiac problems, 1.694 for extracorporeal cardiopulmonary resuscitation; the overall survival rate is 83, 64, and 66%, respectively (ELSO). Although the overall survival is high, there are consistent differences in the incidence rates between the diseases. Since 1979 the survival of ECMO patients with congenital diaphragmatic hernia and sepsis is stable at around 51 and 48%, respectively. In contrast, PPH, RDS, and MAS have a significantly higher survival of 73, 76, and 92%, each. Furthermore, cardiomyopathy and myocarditis have better survival (65 and 50%, respectively), compared to congenital heart disease and cardiogenic shock (44 and 47%, respectively) (ELSO). Nevertheless, the mortality of infants and children with congenital heart disease has reduced in recent decades as a result of the improvement of CPB techniques and myocardial protective strategies (Gilboa et al., 2010; Cheng et al., 2014; Erikssen et al., 2015). Nowadays, although the clinical management of ECMO patients has remarkably progressed over the years, the rate of complications (hemostatic disorders, brain damage, AKI, sepsis) still have a high impact on patients’ outcome (ELSO).

Oxidative stress plays a critical role in the genesis of tissue damage during an ECMO procedure, and newborns are particularly prone to the toxic effects of free radicals, due to the immaturity of their antioxidant systems resulting in lowered detoxification capacity (Comporti et al., 2004; Castellheim et al., 2005; Saugstad, 2005; Mishra et al., 2008; Signorini et al., 2008; Afolayan et al., 2012, 2015; Annagür et al., 2015; Asci et al., 2015; Mokra et al., 2015; Aras-López et al., 2016; Mikolka et al., 2016; Mátyás and Zaharie, 2018; Moore et al., 2018; Perrone et al., 2018). The inflammatory response following the introduction of ECC generates a high amount of ROS contemporarily reducing endogenous antioxidants (Descamps-Latscha, 2001; Goodyear-Bruch and Pierce, 2002; Karu et al., 2010). Various studies associated the exposure to extracorporeal circuits (CPB, ECMO, and CRRT) with the increased oxidative stress that may negatively impact on morbidity and mortality (McDonald et al., 2014a). Redox imbalance is mainly induced in the early stage of ECMO, especially after the CPB, while with prolonged exposure (beyond 24 h) the TAC gradually restores (Wan et al., 1997; Babior, 2000; Desborough, 2000; Clermont et al., 2002; Levy and Tanaka, 2003; Kouchoukos et al., 2012; Chen Q. et al., 2014; Zakkar et al., 2015a,b; Fudulu and Angelini, 2016; Petersen et al., 2018). The modulation of oxidative stress would seem to be of great clinical importance. Indeed, its manipulation through the use of exogenous antioxidants, cardioplegia during CPB, blood priming strategies, or miniaturization of the extracorporeal circuit could mitigate the ROS production (Fudulu and Angelini, 2016). However, data are scant, especially for neonatal ECMO, and are mainly related to pre-clinical evidence. This review focuses on the mechanisms of oxidative stress during neonatal ECMO, involved in the pathophysiology of both short and long-term disorders as MOF, hemostatic derangements up to neurodegenerative, cardiovascular and cancer disease in adults. Lastly, a brief overview of currently available antioxidant strategies is provided (Halliwell and Gutteridge, 2015).

## Redox Biology and Oxidative Stress

Energy is produced by mitochondrial metabolism through glucose, amino acids and lipids combustion in the presence of O_2_ (Semenza, 2009; Vento, 2014). Approximately 98% of O_2_ is reduced to H_2_O by the conventional combustion process, while only 2% of remaining O_2_ loses its electrons with the rear O_2_^-^ and ROS production (Maltepe and Saugstad, 2009; Vento, 2014).

The “old definition” of oxidative stress was referred to conditions in which the balance between ROS production and endogenous antioxidants break down resulting in tissue and cellular injury through lipid peroxidation, protein oxidation, and DNA damage (Schieber and Chandel, 2014). Currently, the “new definition” is much more complex, referring to an imbalance between oxidants and antioxidants in favor of the oxidants, that cause an arrest of redox signaling and/or molecular damage (Sies, 2018). More recently the term “reductive stress” has been introduced, but its exact definition is still unclear (Gutteridge and Halliwell, 2018). It would seem to be due to high levels of reduced GSH and NADPH, unbalancing the redox system to the opposite oxidative extreme, which has shown to play an essential role in the pathogenesis of dilated cardiomyopathy in cases of lamin-associated muscular dystrophy (Margaritelis et al., 2014; Dialynas et al., 2015; Stevens et al., 2017; Bellezza et al., 2018).

**FIGURE 1:**
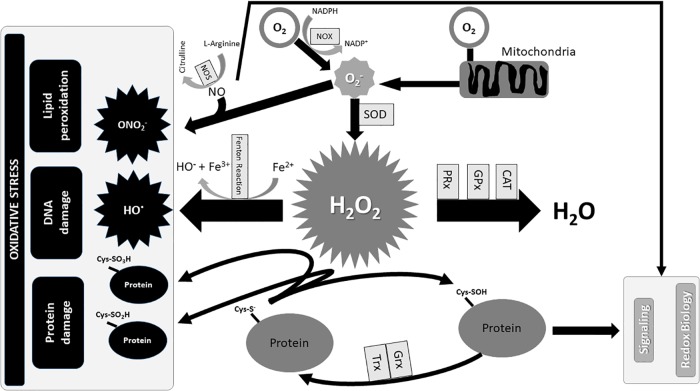
Redox Biology and Oxidative stress. The production of O_2_^-^ mainly occurs with the oxidation of NADPH into NADP+ in the presence of O_2_ and NOX, and partly from an electron’s loss during the combustion of carbohydrates, amino acids, and lipids for the production of energy in mitochondria. Superoxide dismutases (SOD) immediately transforms $O_{2}^{-}$ into H_2_O_2_ which is converted in OH^●^ in the presence of iron, if not degraded by antioxidant enzymes in H_2_O. Furthermore, the excess of $O_{2}^{-}$ reacting with NO produces ON$O_{2}^{-}$, which in turn participates in oxidative damage with OH^●^. Contrarily, the standard production of O_2_^-^ contributes to the homeostasis of the redox signal. CAT, catalase; Cys–S^-^, cysteine thiolate anion; Cys-SO_2_H, cysteine sulfinic acid; Cys-SO_3_H, cysteine sulfonic acid; Cys–SOH, cysteine sulfenic acid; Fe^2+^, ferrous ions; Fe^3+^, ferric ions; GPx, glutathione peroxidases; Grx, glutaredoxin; H_2_O_2_, hydrogen peroxide; NADP+, nicotinamide adenine dinucleotide phosphate oxidated form; NADPH, nicotinamide adenine dinucleotide phosphate reduced form; NO, Nitric oxide; NOS, nitric oxide synthase; NOX, NADPH oxidase; O_2_, oxygen; O_2_^-^, superoxide anion; OH, hydroxyl radicals; ONO_2_^-^, peroxynitrite; PRx, peroxiredoxins; SOD, superoxide dismutases; Trx, thioredoxin.

The O_2_^-^, H_2_O_2_, OH^●^, and ONO_2_^-^ are the most common ROS, produced by aerobic metabolism (Schieber and Chandel, 2014). The intracellular O_2_^-^ arises from the one-electron reduction of molecular O_2_ that is primarily produced by the oxidation of NADPH by NOX enzymes or by electron loss from aerobic respiration in mitochondria (Lambeth, 2004; Brand, 2010; Sahoo et al., 2016). Superoxide anion is rapidly converted by SOD into H_2_O_2_, and its storage is prevented by SODs, which are mainly located in the cytosol and mitochondria. Conversely, the increase in H_2_O_2_ triggers autophagy and cell death (Figure 1; Fridovich, 1997; Chen Y. et al., 2009).

**FIGURE 2:**
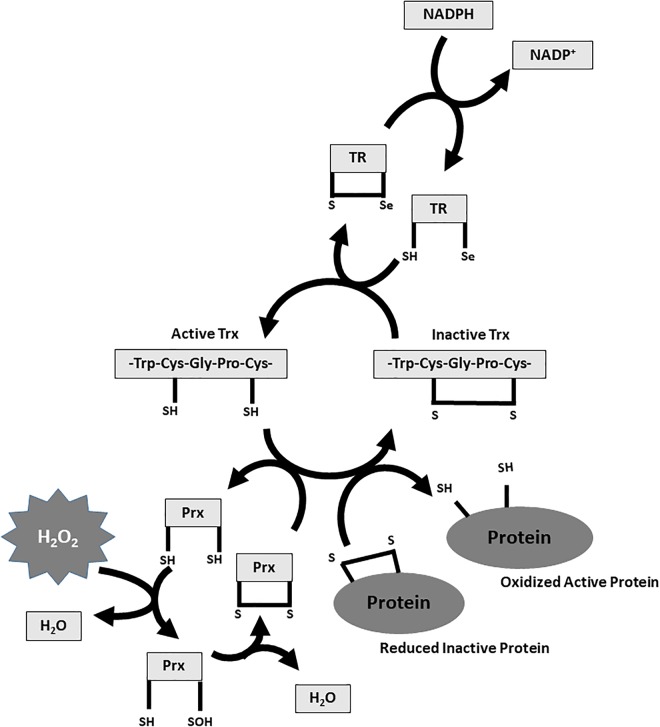
Thioredoxin and Peroxiredoxin system. Reduced Trx-SH catalyzes the reduction of disulfide (s–s) bridges within oxidized cell proteins. This process causes the oxidation of Trx-SH to Trx-disulfide which is subsequently reduced by TR at the expense of NADPH. The TR in its catalytic Trx reduction process uses Se as a cofactor. Reduced PRx-SH promotes the separation of two H_2_O molecules from H_2_O_2_ through a “two-step mechanism.” The peroxidic cysteine sulfhydryl group of a Prx subunit is oxidized to sulfenic acid (–SOH). Subsequently, sulfenic acid condenses with the reduced cysteine group present in the other subunit to form a disulfide bond between the units. This two-step process releases two H_2_O molecules from H_2_O_2_ with the oxidation of PRx-SH to PRx-SOH to PRx-disulfide. The oxidized PRx is in turn reduced by the Trx-SH. H_2_O_2_, hydrogen peroxide; NADP+, nicotinamide adenine dinucleotide phosphate oxidated form; NADPH, nicotinamide adenine dinucleotide phosphate reduced form; PRx, peroxiredoxin; TR, thioredoxin reductase; Trx, thioredoxin.

Cysteine residues within proteins are oxidized by H_2_O_2_ to sulfenic form (Cys–SOH) and reduced to its basic thiolate anion (Cys–S^-^) form by the disulfide reductases Trx and Grx (Winterbourn and Hampton, 2008). The excess of H_2_O_2_ further oxidizes thiolate anions to sulfinic (Cys-SO_2_H) or sulfonic (Cys-SO_3_H) classes. The sulfinic and sulfonic oxidation is irreversible and could lead to permanent protein damage that is prevented by PRx, GPx and CAT (Figures 1, 2; Winterbourn and Hampton, 2008; Finkel, 2012). In contrast, the OH produced by Fenton reaction in the presence of Fe^2+^ has a detrimental effect on lipids, proteins, and DNA (Figure 1; Dizdaroglu and Jaruga, 2012).

Nitric oxide is one of the most important and ubiquitous intracellular messengers that mediates several basic physiological processes including neurotransmission, vasodilatation, angiogenesis and host cell defense (MacMicking et al., 1997; Egginton, 2009; Bogdan, 2015; Philippu, 2016; Kraehling and Sessa, 2017). NO is synthesized from L-arginine by several forms of NOS, and its reaction with O_2_^-^ produces ONO_2_^-^ which alters mitochondrial function and causes apoptosis (Figure 1; Stuehr, 1999; Pacher et al., 2007; Radi, 2013).

### Lipid Peroxidation

Lipid peroxidation, caused by increased ROS and RNS, is linked to many acute and chronic diseases of adulthood such as MOF, hemorrhagic and septic shock, ARDS, acute lung injury, cardiovascular pathology requiring surgery, aging, atherosclerosis, Parkinson, Alzheimer, diabetes mellitus, cataracts and rheumatoid arthritis, and several diseases of the neonatal period (Takeda et al., 1984; Hammond and Hess, 1985; Bertrand et al., 1989; Richard et al., 1990; Quinlan et al., 1996; Carpenter et al., 1998; Lamb et al., 1999; Motoyama et al., 2003; Comporti et al., 2004; Quinlan et al., 2004; Fläring et al., 2005; Laplace et al., 2005; Niki et al., 2005; Saugstad, 2005; Huet et al., 2007; Signorini et al., 2008; Giustarini et al., 2009; Briones and Touyz, 2010; Giacco and Brownlee, 2010; Khaper et al., 2010; Romano et al., 2010; Barrera et al., 2018; Maiorino et al., 2018; Mátyás and Zaharie, 2018; Moore et al., 2018; Orban and Singer, 2018; Perrone et al., 2018; Romano et al., 2018).

Lipid peroxidation is the oxidative decay of Puffs, which involves the abstraction of hydrogen from a carbon molecule with O_2_ insertion and consequent production of lipid peroxyl radicals and hydroperoxides (Tappel, 1970; Yin et al., 2011; Al-Dalaen and Al-Qtaitat, 2014). Hydroperoxides are very unstable and easily decompose into MDAs and 4-hydroxy-2-nonenal, while peroxidation of arachidonic acid produces isoprostanes (Montuschi et al., 2004; Lakshminrusimha et al., 2006; Al-Dalaen and Al-Qtaitat, 2014). The response of the cell membrane to lipid peroxidation depends on the degree of peroxidation achieved. If lipid peroxidation rate is low, the damage is repaired by anti-oxidant defense systems. On the other hand, if the lipid peroxidation rate is high, the tissue self-repairing capacity is lost, and apoptosis or programmed cell death is induced (Chaudhary et al., 2010; Ayala et al., 2014).

### Oxidative Protein Damage

Protein oxidation damage does affect not only the cell membranes but also the enzyme system because it is exclusively constituted by proteins (Goodyear-Bruch and Pierce, 2002). The amino acid fragmentation, the following generation of cross-links between proteins and the oxidation of the body of the protein molecule with loss of specific (contractile, enzymatic, and transport) function is caused by ROS and RNS (Dalle-Donne et al., 2003a,b; Giustarini et al., 2009). Protein Carbonyl groups (aldehydes and ketones) are produced generally by the direct oxidation of proline, arginine, lysine, and threonine residues, or by oxidative cleavage of the protein backbone, and indirectly by lipid peroxidation or reaction of reducing sugars or their oxidation products (Dalle-Donne et al., 2003a, 2006). The damaged proteins, in turn, contribute to many disorders and diseases (Dalle-Donne et al., 2003a). Oxidized or aldehyde-modified proteins would be perceived as “foreign proteins” by the immune system, with the production of self-antibodies in rheumatoid arthritis, lupus, and scleroderma in adults (Mantle et al., 1999; Dimon-Gadal et al., 2000; Renke et al., 2000; Dalle-Donne et al., 2003a; Halliwell and Gutteridge, 2015). Moreover, always in adults, the increase of oxidized proteins has been associated with multiple diseases, like cancer, heart failure, pre-eclampsia, ARDS, Parkinson, Alzheimer, and sepsis (Floor and Wetzel, 1998; Lenz et al., 1999; Winterbourn et al., 2000; Butterfield et al., 2001; Zusterzeel et al., 2001; Abu-Zidan et al., 2002; Castegna et al., 2002). In the neonatal period, the oxidation of proteins plays a key role in some diseases such as intraventricular hemorrhage, retinopathy of prematurity, bronchopulmonary dysplasia, NEC (Gladstone and Levine, 1994; Ramsay et al., 2001; Perrone et al., 2009, 2010, 2014, 2018; Couroucli, 2017).

### Oxidative DNA Damage

Oxidative DNA damage is generated by high OH^●^ concentrations (Cooke et al., 2003). ROS can damage both purines and pyrimidines in DNA and in the free cell nucleotide pool and generates 8-hydroxy-2′-deoxyguanosine that can be used as a biomarker of the injury (Suliman et al., 2003; Bayr, 2005; Li et al., 2014). Despite the damage, the cell continues the replication process, replicating the damage and inducing the new cells to apoptosis (Roos and Kaina, 2006). Furthermore, oxidative DNA damage would be associated with other diseases such as sepsis, carcinogenesis, neurodegenerative diseases, cardiovascular diseases, and aging (Cooke et al., 2003; Niki et al., 2005; Giustarini et al., 2009; Halliwell and Gutteridge, 2015; Bahar et al., 2018; Gutteridge and Halliwell, 2018; Klaunig and Je, 2018).

## Oxidative Stress and ECMO

To date, evidence of oxidative stress during ECMO are limited and are mainly related to pre-clinical studies (Möller et al., 1993; Trittenwein et al., 1999). Möller et al. (1993) observed a reduction of the antioxidant capacity of SOD and GPx on a lamb ECMO model, while increased lipid peroxidation was demonstrated on a rabbit model of ECMO (Trittenwein et al., 1999). One study on pediatric patients described two different oxidative stress patterns during ECC; in non-survivors, lipid peroxidation augmented during the first 96 h of ECMO whereas it reached a plateau after 24 h in the survivor’s group (Hirthler et al., 1992).

No data are currently available on critically ill neonates undergoing respiratory ECMO. Despite this gap of knowledge, previous evidence, mainly derived from studies on CPB and critically ill patients, suggested that oxidative stress is a relevant contributor to morbidity and mortality (Möller et al., 1993; Trittenwein et al., 1999; Brix-Christensen et al., 2001, 2002; Goodyear-Bruch and Pierce, 2002; Levy and Tanaka, 2003). ECMO shares with both CPB and critical illness common potential triggers for increased ROS generation and redox unbalance: exposure to the extracorporeal circuit and related SIRS, transfusion burden, hyperoxia, CRRT, hemolysis, and sequestration of antioxidants into the ECMO circuit (McDonald et al., 2014a). We describe hereafter the possible role of these triggers in the pathogenesis of oxidative stress-related morbidity during ECC.

### ECMO and Inflammation

A SIRS-like syndrome is typically observed from the first hours after starting an ECMO procedure, as a consequence of the blood exposure to the exogenous surface of the extracorporeal device (Davies and Hagen, 1997). Despite the use of biocompatible equipment, the interaction between biological fluids and the circuit activates both the coagulation cascade and the host inflammatory response (Tayama et al., 2000; Palatianos et al., 2003; Wiesenack et al., 2004; Thiara et al., 2010). The humoral and cell-mediated immune response triggers the release of pro-inflammatory cytokines (IL-1β, TNFα, and IFNβ), leading to endothelial damage, disrupted microcirculation and multi-organ dysfunction, in case of suboptimal compensatory anti-inflammatory response (Hocker et al., 1991; Hirthler et al., 1992; Depuydt et al., 1993; Plötz et al., 1993; Fortenberry et al., 1996; Adrian et al., 1998; Graulich et al., 2002; Golej et al., 2003; Mildner et al., 2005; Adib-Conquy and Cavaillon, 2009; Mcilwain et al., 2010; He et al., 2014; Shi et al., 2014; Rungatscher et al., 2015; Wang et al., 2015). The redox biology regulates the homeostasis of innate and adaptive immune response through ROS production. Many concurrent factors (hyperoxia, blood products, sepsis, mechanical ventilation) contribute to the increased ROS generation, thus promoting an overwhelming release of pro-inflammatory cytokines (IL-1β, TNFα, and IFNβ) and eventually the SIRS-like (Wiesenack et al., 2004; Cruz et al., 2007; Chen and Nuñez, 2010; Iwasaki and Medzhitov, 2010; Bulua et al., 2011; West et al., 2011; Mittal et al., 2014).

Furthermore, the intrinsic and extrinsic coagulation cascade could be directly activated by the contact of blood with the circuit surface or lesion of endothelium, or indirectly by ROS mediation (Davie et al., 1991; Görlach, 2004, 2005; Herkert et al., 2004; Mackman et al., 2007; Oliver, 2009; Fall et al., 2015). The intrinsic coagulative pathway would be activated exclusively during the respiratory ECMO with low thrombin generation; while the release of high amount of TF and air-blood interface would stimulate both the intrinsic and extrinsic way with high thrombin generation during the CPB (Levy and Tanaka, 2003; Oliver, 2009; Kraft et al., 2015). Indeed, TF binds to Factor VII, converting it into Factor VIIa and activating the common coagulation cascade (Davie et al., 1991; Levy and Tanaka, 2003; Mackman et al., 2007; Oliver, 2009). The intrinsic pathway is activated through the conversion of Factor XII into Factor XIIa within 10 min from the beginning of the extracorporeal procedure and is associated with an increase in Bradykinin, Kallikrein, and activation of Factor IX (Wendel et al., 1999; Millar et al., 2016). Bradykinin stimulates NO, TNFα, and IL-10 production, while Kallikrein directly activates neutrophils (Rodell et al., 1995; Wachtfogel et al., 1995). Factor IXa induces the activation of Factor X which in turn causes the conversion of prothrombin into thrombin and fibrinogen in fibrin resulting in thrombus formation (Millar et al., 2016). Thrombin stimulates the endothelial production of P-selectin, E-selectin, and platelet activating factor, thus increasing the adherence and activation of neutrophils and the expression of proinflammatory cytokines (Prescott et al., 1984; Zimmerman et al., 1985; Kaplanski et al., 1997; Levy and Tanaka, 2003). Additionally, thrombin stimulates the production of ROS in endothelial and smooth muscle cells, by stimulating NOX and by up-regulating the expression of NOX sub-unit p22phox in a kinase-dependent manner (Görlach et al., 2002; Djordjevic et al., 2005).

Oxidative stress induces platelets activation and aggregation which, in turn, increases ROS production. Neutrophils participate in the amplification of the immune response; although multifocal, the activation of neutrophils occurs mainly in the oxygenator; their margination in the artificial lung, observed both in animal models and in newborns, contribute to the deterioration of the artificial membrane during ECMO (Plötz et al., 1993; Fortenberry et al., 1996; Kotani et al., 2000; Brix-Christensen et al., 2002; Mcilwain et al., 2010; Rungatscher et al., 2015).

**FIGURE 3:**
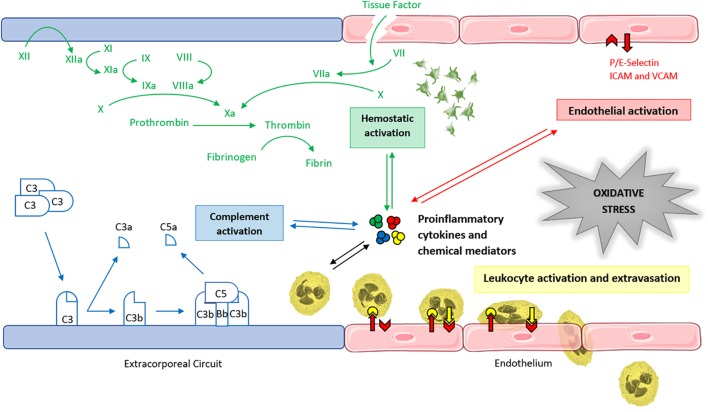
Main determinants of ECMO-induced oxidative stress. Graphical representation of the interaction between the coagulation cascade, the immune response, and the endothelium in the multifactorial amplification process of the ECMO-related redox unbalance. ICAM, intercellular adhesion molecule. VCAM, vascular cell adhesion molecule.

In conclusion, the mechanism of ECMO-induced oxidative stress is a multifactorial process, in which the interaction between the immune response, the coagulation cascade, and endothelial cells amplifies ROS generation (Figure 3).

### ECMO and Blood Transfusion

Blood products have been associated with increased morbidity and mortality in critically ill patients (Koch et al., 2008; Wang et al., 2012; Ghirardello et al., 2017). Transfusion-related oxidative stress increases with the volume, and the number of PRBC transfused (Shohat et al., 1983; Silvers et al., 1998; Dani et al., 2001; Collard et al., 2004). Rosa et al. (2011) observed that blood transfusions were associated with a significant increase in oxidative stress and a reduction of IL-6 concentration in critically ill adults. The transfusion of aged PRBC was associated with an increase of oxidative stress, as a consequence of the reduced Se content in older PRBC. Se is an essential trace element, which is incorporated into selenoproteins, some of which have antioxidants function (i.e., GPx) (McDonald et al., 2014b). In premature newborns, RBC transfusions were associated with an increase of MDA, a lipid peroxidation product, both in the bronchoalveolar lavage fluid and in urine (Wardle et al., 2002). This data suggested that RBC transfusion could increase pulmonary oxidative damage and worse prognosis (Collard et al., 2005).

Extracorporeal membrane oxygenation, as well as CPB or exchange transfusion, are procedures that expose patients to a significant increase in PRBC transfusions and therefore to oxidative stress (McCoy-Pardington et al., 1990; Luban, 1995; Butch et al., 1996; Ang et al., 2009). Newborns receive an average of 60 ml/kg/day of PRBC during an ECMO procedure (Stiller et al., 2004). This high blood requirement depends on (1) priming of the ECMO circuit with blood, to avoid acute hemodilution, (2) frequent blood samples for coagulation tests, (3) hemolysis-related anemia due to centrifugal pumps and, occasionally, (4) spontaneous bleeding from cannulation sites and surgical wounds. According to the ELSO registry, these latter events represents about 19% of all hemorrhagic complications during neonatal respiratory ECMO, impacting on survival (ELSO). Overall, the mortality rate is increased among neonates exposed to large volumes of PRBC transfusions (Smith et al., 2013; Fiser et al., 2014; Ghirardello et al., 2017). Dos Santos et al. observed a 27.8% mortality in critically ill infants exposed to transfusions during the hospital stay, with an increased relative risk of death of 1.49 in those infants who received at least one transfusion compared to controls (Dos Santos et al., 2011). In a cohort of extremely low birth weight infants, the incidence of mortality was 24% in patients transfused compared to 8.5% in the control group (Ghirardello et al., 2017). Similarly, Smith and co-authors observed a 24% increase in the odds of hospital death associated with every 10 ml/kg/day of PRBC transfusion in patients during respiratory ECMO (Smith et al., 2013).

In addition to PRBC, newborns on ECMO require approximately 25 ml/kg/day of PCs (Stiller et al., 2004). Platelet count is reduced by 40–50% during ECMO, partly due to the activation of the coagulation cascade (Nawab and Williams, 2017). Moreover, PCs are a source of ROS, especially $O_{2}^{-}$, which modulate platelet activation and aggregation (Krötz et al., 2002; Begonja et al., 2005). The increase in ROS, whose impact on the patient’s oxidation state is still unknown, is mainly related to the storage process (Sonego et al., 2017).

### ECMO and Hemolysis

ECMO-related hemolysis is a common phenomenon among pediatric patients and represents an independent predictor of mortality (Omar et al., 2015; Dalton et al., 2018). It consists of the RBCs lysis with following release of hemoglobin into the plasma (Rother et al., 2005). The shear stress is the main causative mechanism for hemolysis during mechanical support, and some risk factors have been identified, such as the negative inlet pressure, pump speed, pump and oxygenator type, cavitation and priming solution (Steinhorn et al., 1989; Lou et al., 2014; Rother et al., 2005). In addition, oxidative stress is a determinant of hemolysis (Boyle et al., 1997; Walski et al., 2018). Lipid peroxidation and protein damage of cell membranes from ROS cause enzymatic inactivation, depolarization of the cell membrane and a change in its permeability, resulting in cell lysis (Matés et al., 1999; Kirkham and Rahman, 2006). The mechanical and oxidative stress during ECMO leads to the release of the PFHb, which is cleared by hemoglobin scavengers (Dalton et al., 2018). However, in case of severe hemolysis, the hemoglobin-scavenging mechanisms become saturated, resulting in hemoglobinemia and hemoglobinuria (Rother et al., 2005). The PFHb may exert either a direct toxic effect (i.e., renal damage) or may cause endothelial dysfunction, by binding NO. The NO consumption leads to increased systemic and pulmonary resistance, platelet activation and aggregation, endothelial adhesion, fibrin deposition and thrombin formation (Rother et al., 2005).

Moreover, the shear stress induces procoagulant conformational changes of the pVWF, which interacts with circulating platelets enhancing their adhesion and aggregation properties (Cruz et al., 2018).

Furthermore, high levels of PFHb inhibit the cleavage of pVWF by ADAMTS-13, contributing to increased clotting within the mechanical devices (Da et al., 2015). Eventually, hyperbilirubinemia and iron release constitute additional drivers of the hemolysis-induced morbidity and mortality (Davis et al., 1999; Kaplan et al., 2014). Besides hemolysis, the mechanical stress may hesitate into sublethal RBCs damage, leading to increased fragility and decreased deformability of RBCs (Kameneva et al., 1999). Their consequent failure to enter small capillaries causes end-organ dysfunction, through the impairment of tissue oxygenation and microcirculation (Watanabe et al., 2007). Based on the concepts mentioned above, ECMO providers should monitor the hemolysis, as it is strictly associated with increased mortality and morbidity (kidney injury, vascular impairment, need for PRBC and PCs transfusion, thrombosis).

Moreover, efforts should focus on the technological progress, because of specific components of the circuit act as main drivers of the ECMO-induced hemolysis, such as the oxygenator (Williams et al., 2015). The use of smaller pediatric oxygenators was associated with greater shear stress and, hence, increased hemolysis, when compared to larger adult oxygenators (Williams et al., 2015). Although the relative contribution of each circuit component on the hemolysis and related oxidative stress is unknown, the use of a centrifugal pump and small size connectors should be considered as potential sites of increased hemolysis (Lou et al., 2014; Williams et al., 2015). Furthermore, the use of red-to-infrared radiation (R/NIR) to stabilize erythrocyte membranes with increased resistance to oxidative stress seems promising (Itoh et al., 2000; Komorowska et al., 2001; Chludzińska et al., 2005; Walski et al., 2015, 2018).

Technology advances should target the development of new, more bio-compatible, circuit-blood interfaces in order to reduce hemolysis and oxidative stress.

### ECMO and Hyperoxia

The antioxidant capacity of tissues is overtaken with O_2_^-^ production during hyperoxia. Oxygen toxicity in the perinatal period is well established, and many efforts have been made to define safety targets during neonatal resuscitation and care (Tin and Gupta, 2007; Auten and Davis, 2009; Finer and Leone, 2009; Askie et al., 2011; Saugstad et al., 2012; Vento et al., 2012; Schmidt et al., 2013; Saugstad and Aune, 2014; Manja et al., 2015).

Nevertheless, hyperoxia remains a significant driver for radical O_2_ species generation, especially during ECC (Cashen et al., 2018). Hyperoxia is frequent during cardiac surgery in CPB and seems to play a key role in the development of oxidative stress as a consequence of post-ischemic reperfusion (Cavarocchi et al., 1986; Börgermann et al., 2007). The clamp of the aorta induces heart and lung ischemia while declamping generates reperfusion, and either action produces an inflammatory response. On-pump cardiac surgery is associated with increased oxidative stress if compared to off-pump surgery (Matata et al., 2000; Cavalca et al., 2006). The post-ischemic reperfusion, rather than hyperoxia *per se*, seems to play a role; there is evidence from pediatric cardiac surgery that isolated hyperoxia does not increase oxidative stress and inflammation if compared with normoxia (Hövels-Gürich et al., 2002; Caputo et al., 2005, 2009). Although ECMO does not directly cause ischemia/reperfusion, patients requiring ECMO experience prolonged periods of pre-ECMO hypoxia causing an increase in oxidative stress (Crimi et al., 2006; Hayes et al., 2013; Chen Q. et al., 2014). ROS generation following hyperoxia is significantly exaggerated when hyperoxia is subsequent a period of hypoxia, due to the dysfunction of the post-ischemic mitochondrial electron transport chain (Neumar, 2011). Sznycer-Taub et al. (2016) identified hyperoxia within 48 h of ECMO following cardiac surgery as an independent risk factor for mortality at 30 days post-operatively, while Trittenwein et al. (1999) observed how VV-ECMO more than VA-ECMO would increase pulmonary lipid peroxidation following endotoxemia-induced hypoxia. Lipid peroxidation of the lung is a known consequence of sepsis and inflammation, but it has also been described as a consequence of tissue reperfusion (Demling et al., 1991; Horton and Walker, 1993; Christie et al., 1994; Chow et al., 2003). In the study by Trittenwein et al. (1999) hyperoxic reperfusion of the hypoxic lung following VV-ECMO, caused a significant increase of plasma MDA as an index of lipid peroxidation, higher than that observed in VA-ECMO where the hyperoxic blood supply to the lung is less.

More recently, similar results have been observed by Cashen et al. (2018) that showed higher in-hospital mortality among hyperoxic patients submitted to ECMO compared to normoxics, ascribing the high mortality to the effects of ROS on poor cardiac output rather than the SIRS-like syndrome, hemostatic activation and MOF.

### ECMO and Continuous Renal Replacement Therapies

Oxidative stress is a common condition in kidney diseases (Witko-Sarsat et al., 1996; Hasselwander and Young, 1998); it is induced by renal pathology itself, reduced antioxidant intake associated with malnutrition, CRRT with the loss of antioxidant molecules during filtration and the contact of blood with CRRT circuit (Wolf et al., 1951; Hasselwander and Young, 1998; Cano, 2001; Sosa et al., 2006; Varan et al., 2010; Libetta et al., 2011; Coombes and Fassett, 2012; Ozbek, 2012). The SIRS-like syndrome, which typically occurs in the early hours of ECMO, is associated with hypotension, oligo-anuria, decreased lung compliance, pulmonary edema, increased plasma concentrations of pro-inflammatory cytokines and neutrophils’ activation (Hocker et al., 1991; Davies and Hagen, 1997). AKI and FO complicate neonatal ECMO runs in more than half of cases, with a negative impact on survival rates (Heiss et al., 1987; Weber et al., 1990; Swaniker et al., 2000; Gadepalli et al., 2011; Zwiers et al., 2013). Therefore, hemofiltration and hemodialysis have become a standard of care in the management of AKI during ECMO (Heiss et al., 1987; Meyer et al., 2001; Paden et al., 2011; Chen H. et al., 2014). During CRRT, inflammation is activated by an increase of ROS and inflammatory cytokines (TNFα, IL-1, and IL-6) (Tarakçıoğlu et al., 2003; Borazan et al., 2004) and a reduction of antioxidant proteins as hydrosoluble vitamin C and uric acids (Hegbrant and Hultkvist, 1999; Yang et al., 2006; Libetta et al., 2011). The use of biocompatible filtration systems by using vitamin E-coated filters reduces the oxidative stress linked to the contact of blood with exogenous materials (Yang et al., 2014). Selewski et al. (2012) suggested an early filtration treatment to anticipate FO, and reduce mortality rate. As reported by ELSO registry, about 33% of neonatal respiratory ECMO and 38% of cardiac ECMO require renal replacement therapy (ELSO). As a result of the combination of two ECC systems, the hemolysis-related oxidative stress would increase (Chen H. et al., 2014). The in-line use of CRRT would limit the ECMO-related inflammation by reducing the concentrations of TNFα, IL-1β, IL-6, and IL-8 (Jialiang et al., 2014).

### ECMO Post-cardiopulmonary Bypass

Adult patients with cardiovascular diseases and neonatal patients with congenital cyanotic heart diseases have an increased level of oxidative stress, even before heart surgery, if compared to the healthy population (Ercan et al., 2009; Giustarini et al., 2009; Karu et al., 2010; Small et al., 2012).

Moreover, cardiac surgery is always associated with both systemic inflammation and a variable degree of oxidative stress, which might contribute to post-operative complications, failure of single and multiple organs and mortality (Kim et al., 2008; Billings et al., 2011; Antoniades et al., 2012; Plicner et al., 2014; Wu et al., 2015). Plicner et al. (2014) observed that the increase of asymmetric dimethylarginine and 8-iso-prostaglandin F_2α_ was associated with an unfavorable outcome of patients undergoing coronary artery bypass grafting. Similarly, Antoniades et al. (2012) has shown how the increase of O_2_^-^ and ONO_2_^-^ concentration was closely related to atrial fibrillation, need for inotropic drugs and lengthening of hospital stay during post-cardiac surgery.

The main cause of oxidative stress during cardiac surgery is the exposure to the foreign surface of the extracorporeal circuit, hypothermia, and circulatory arrest associated with systemic inflammation, ischemia-reperfusion injury, altered hemostatic status (Prasad et al., 1992; Edmunds, 1998; Clermont et al., 2002; Levy and Tanaka, 2003; Ulus et al., 2003; Gessler et al., 2004; Karu et al., 2010). Approximately 3–5% of patients undergoing congenital cardiac surgery require post-CPB extracorporeal support, due to the weaning failure following myocardial dysfunction, low cardiac output state, cardiac or respiratory arrest, pulmonary hypertension, or shunt occlusion (Aharon et al., 2001; Salvin et al., 2008; Mascio et al., 2014). The long duration of ECMO post-CPB might impair the organ function, thus predisposing to an increased risk of complications (i.e., sepsis) and mortality (Duncan et al., 1999; Chaturvedi et al., 2004; Salvin et al., 2008; Kumar et al., 2010; Rood et al., 2011; ELSO, 2018).

## Clinical Relevance of Oxidative Stress During Neonatal ECMO

**FIGURE 4:**
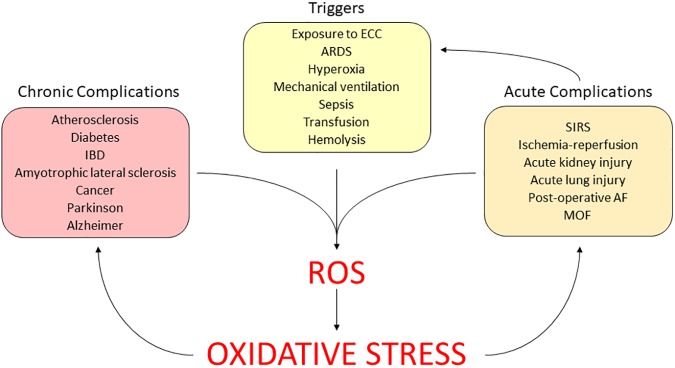
Clinical relevance of ECMO-induced oxidative stress. AF, atrial fibrillation; ARDS, acute respiratory distress syndrome; ECC, extracorporeal circuit; IBD, intestinal bowel disease; MOF, multi-organ failure; SIRS, systemic inflammatory response syndrome.

The role of continuing oxidative stress has been established in the long term, in a variety of chronic diseases, such as diabetes mellitus, atherosclerosis and neurodegenerative illness (Haidara et al., 2006). Currently, the interest is moving toward the effects of the redox unbalance in the acute illness (Bar-Or et al., 2015). Here, we summarize the available evidence in relation to the role of the oxidative stress in critical conditions, which often occur during ECMO (Figure 4).

### Sepsis and Multi-Organ Failure

A wealth of studies has shown that the normal redox balance is altered in septic shock and multiple organ failure, due to both an increase in $O_{2}^{-}$ derived free radicals and a decrease in the plasma antioxidant potential (Ogilvie et al., 1991; Goode et al., 1995). Adult septic patients have higher plasma CAT and SOD if compared to healthy controls (Warner et al., 1995). Moreover, non-survivors of sepsis have shown higher levels of SOD and a reduced plasmatic antioxidant status, which was associated with unfavorable outcomes (Cowley et al., 1996; Guerreiro et al., 2010). The sepsis-related MOF, which remains the major cause of death in intensive care units, is associated with oxidative stress and mitochondrial dysfunction (Brealey et al., 2002; Galley, 2011). Two antioxidants targeting the protection of mitochondria (MitoQ and MitoVitE) through the reduction of oxidative stress have been beneficial in a rat model of acute sepsis (Lowes et al., 2013). Other potential oxidative-induced mechanisms for MOF development have been proposed, as a result of the anti-oxidant impairment secondary to GSH and Se depletion, suggesting a positive effect of Se supplementation (Brealey et al., 2002; Manzanares et al., 2012; Broman et al., 2018).

### Hemostatic Derangement and Anticoagulation Management

Maintaining the hemostatic homeostasis during ECMO is extremely challenging and, despite technical advances, bleeding and thrombosis remain serious complications (Murphy et al., 2015). The hemostatic perturbation during ECMO is a multifactorial process, and the redox imbalance is one of the main contributing factors. Besides the dilutional coagulopathy, which depends on priming compositions and affects both clotting factors and platelets, the shear stress has a key role as it induces platelet dysfunction, fibrinolysis activation, acquired von Willebrand syndrome and intravascular hemolysis (Görlach et al., 2002; Görlach, 2004, 2005; Fall et al., 2015). As previously mentioned, the oxidative stress is strictly associated with the shear stress, resulting in endothelial activation (see section “ECMO and Inflammation”) and enhanced hemolysis (see section “ECMO and Hemolysis”) (Görlach et al., 2002; Görlach, 2004, 2005; Herkert et al., 2004; Djordjevic et al., 2005; Millar et al., 2016).

Moreover, the exposure to the exogenous circuits triggers a SIRS-like syndrome, which is associated with the ROS generation, the release of pro-inflammatory cytokines, the activation of the intrinsic coagulation pathway with a potential procoagulant effect (Millar et al., 2016). The clot formation triggers the consumption of coagulation factors and platelets, thus increasing the risk of disseminated intravascular coagulopathy and bleeding (Millar et al., 2016). The anticoagulation management should be modulated, by taking into account the drivers of hemostatic unbalance mentioned above.

### Biomarkers of the Redox State

Based on the relevant clinical implication of the redox unbalance during ECMO, the identification of diagnostic markers would help in the detection, monitoring, and follow-up of the redox potential in ECMO neonates. However, the profile of biomarkers of both oxidative stress and antioxidant defenses during neonatal ECMO has not been characterized yet. Evidence can be derived from previous pre-clinical and clinical studies, which evaluate the pro-oxidative potential during and after the exposure to an extracorporeal circuit (i.e., CRRT, CPB) (Takouli et al., 2010; McDonald et al., 2012). As most of the research has been performed on the adult population, the evaluation of the redox status in neonatal systemic diseases (i.e., septic shock) may provide further insights into the age-related antioxidant deficiency (Batra et al., 2000). Among potential pro-oxidant biomarkers, the d-ROMs have been evaluated in a dialyzer, while the MDA has been applied in a porcine ECMO model (Takouli et al., 2010; Chen Q. et al., 2014).

Additionally, as nitro-oxidative stress is involved in a large variety of pathological mechanisms, the biomarkers validated for the detection of nitrosation and nitration would be of interest (Cipak Gasparovic et al., 2017). However, their application in the extra-corporeal setting is still lacking. The anti-oxidant response may be evaluated through the detection of the TAC, SOD, and GSx activity (Takouli et al., 2010; McDonald et al., 2012). Moreover, the reduction of key trace elements, such as Se, copper, and zinc might suggest a reduced anti-oxidant activity (McDonald et al., 2012). In clinical practice, the oxidative stress may be indirectly presumed through the monitoring of inflammatory markers, such as the increased levels of IL-6 and IL-8 or by evaluating the hemolysis with the PFHb levels (Rother et al., 2005; Takouli et al., 2010; Cruz et al., 2018).

## Strategies to Reduce Oxidative Stress: Update on Antioxidants

The ROS production is regulated by intra and extracellular antioxidant systems, which are impaired during the perinatal period and critical illness (Manzanares et al., 2012; McDonald et al., 2012). ECMO further complicates the issue, by adding specific sources for redox state unbalance (Manzanares et al., 2012; McDonald et al., 2012, 2014a; Jialiang et al., 2014; Millar et al., 2016; Bocca et al., 2017). The antioxidant system of the newborns is immature with both a reduction of antioxidant enzymes (SOD, CAT, and GPx) and non-enzymatic antioxidants (vitamin E, β-carotene, melatonin, ceruloplasmin, transferrin, coenzyme Q) while ascorbic acid and bilirubin are present only for a short period after birth (Gopinathan et al., 1994; Gitto et al., 2009; Bocca et al., 2017). Free iron levels are significantly higher than in adults, causing an increased Fenton reaction, which in turn stimulates the production of OH^●^ (Saugstad, 2005). Furthermore both the SIRS-like and the sequestration into the ECMO/hemofilter circuit reduce the level of trace elements (copper, manganese, zinc, iron, Se as cofactors for SOD, GPx, and CAT function) and non-enzymatic antioxidants (albumin, uric acid, vitamins C and E) (Cano, 2001; Yamamoto et al., 2003; Yamawaki and Berk, 2006; Berndt et al., 2007; Tonelli et al., 2009; Hoffmann et al., 2011; Visser et al., 2011; Manzanares et al., 2012; Wołonciej et al., 2016; Ciapetti et al., 2018).

Based on the paucity of data regarding patients in ECMO both neonatal and adult, we will illustrate previous data regarding the use of anti-oxidants in critically ill adult patients undergoing surgery, CRRT or CPB. Moreover, taking into account the peculiarity of the perinatal period, we will mention some data on anti-oxidant strategies studied in neonatal conditions where oxidative stress is one of the pathogenic mechanisms, such as sepsis or NEC (Ozdemir et al., 2012; Poggi and Dani, 2018) (Supplementary Table S1). Among others, vitamin A, vitamin E, vitamin C, SOD, and NAC have been studied to reduce tissue damage mediated by oxidative stress with contrasting results (Tyson et al., 1999; Suresh et al., 2001; Wardle et al., 2001; Brion et al., 2003; Darlow et al., 2016).

Antioxidants have two main mechanisms of action: the prevention, inactivating the free radical present in the systems and the scavenging of the active radical, suppressing the chain initiation or breaking the chain propagation. They may exert their effect by many actions including electron donation, hydrogen donor, metal ion chelation, co-antioxidants, radical scavenger, peroxide decomposer, singlet oxygen quencher, enzyme inhibitor or by gene expression regulation (Lobo et al., 2010).

*Selenoproteins* contribute to the function of many enzymes among which GSx, TRs, and methionine sulfoxide reductase and seem to protect the cardiovascular system against oxidative stress (Rotruck et al., 1973; Kim and Gladyshev, 2004; Hoffmann and Berry, 2005; Berndt et al., 2007; Hoffmann et al., 2011). Based on the reduction of Se levels in critically ill patients or after cardiac surgery, Se supplementation has been investigated in this contexts with contradictory results (Manzanares et al., 2012; Stoppe et al., 2013; Broman et al., 2018). To date, there are no data to support its extended use (Allingstrup and Afshari, 2015; Manzanares et al., 2016). Although not conclusive, neonatal supplementation with oral or parenteral Se in the first weeks of life to preterm infants led to a reduction in the proportion of infants having one or more episodes of sepsis (Darlow and Austin, 2003). The administration of pre and post-operative *vitamin C* in cardiac surgical adults has led to a reduction of post-operative atrial fibrillation, which seems to be associated with increased atrial oxidative stress and ONO_2_^-^ formation (Carnes et al., 2001).

Moreover, supplementation with ascorbic acid caused a reduction of plasma levels of oxidative stress during hemodialysis and restoration of endothelial function in critically ill patients (Eiselt et al., 2001; Wilson, 2009).

Similarly, *vitamin E*-coated dialysis membranes used in adult patients on hemodialysis led to a decrease in reactive oxygen metabolites and derivatives, an increase of TAC and superoxide dismutase (Takouli et al., 2010). In addition, a decrease in post-operative atrial fibrillation has been documented after the supplementation of pre and post-operative *NAC* in surgical adult patients (Ozaydin et al., 2008). Ozdemir et al. (2012) have shown a beneficial effect of NAC in a murine NEC model in terms of reduced levels of oxidative stress (MDA) and inflammation (TNFα), with increased antioxidant activity of the SOD. Also concerning the supplementation of *glutamine*, data are not conclusive. Indeed, previous studies showed that infusion of glutamine during and for 3 days after cardio-surgery in adult patients led to an increase of glutamine blood levels and GSH activity without clinical improvement (Engel et al., 2009). Enteral glutamine either alone or in combination with arginine has been tested in healthy newborn rats showing a reduction of oxidative stress. These results may suggest a potential benefit of glutamine and arginine supplementation in the prevention of NEC in premature neonates with insufficient oxidative resistance (Kul et al., 2009). The evidence is building on the neonatal use of the ROS scavenger *melatonin*. Its supplementation in newborns with sepsis within the first 12 h after diagnosis has led to a reduction of lipid peroxidation products (MDA) and improvement of clinical outcomes (Gitto et al., 2001). The post-operative administration of melatonin reduced the proinflammatory cytokines and NO levels in newborns undergoing surgery (Gitto et al., 2004).

Moreover, a reduction of late-onset sepsis risk ratio has been shown in newborn supplemented with *lactoferrin* and a reduction of the risk of antibiotic treatment failure in septic newborns exposed to *zinc* (Bhatnagar et al., 2012; Pammi and Suresh, 2017). Additional promising agents are the *apocynin*, an NOX inhibitor applied in the animal model of stroke and brain injury induced by ischemia-riperfusion, and the *α-lipoic acid*, a GSH-mediated antioxidant used in adults undergoing surgery during ECC (Packer et al., 1997; Kahles et al., 2007; Lambeth et al., 2008; Aly et al., 2009; Chen H. et al., 2009; Choi et al., 2010; Xia et al., 2010; Simonyi et al., 2012; Uyar et al., 2013). Novel antioxidant molecules, such as the ROS scavenger *edaravone*, showed promising results. In a pre-clinical model of neonatal sepsis the administration of edaravone 30 min after the injury has led to both a biochemical and clinical improvement (Kato et al., 2009). Other agents like *hyperbaric oxygen and medical ozone* have been suggested to reduce oxidative stress by enhancing the antioxidant system. The technique based on the use of O_2_/ozone mix was previously applied in CRRT with promising results, even if its mechanism of action remains still unveiled (Bocci et al., 1999, 2001; Bocci and Paolo, 2004; Di Paolo et al., 2005). Data on the use of ozone in ECMO are lacking and might be a future area of interest. Lastly, it is worth mentioning the potential role of hMSCs in ischemia/reperfusion-induced lung injury, by acting through anti-oxidant, anti-inflammatory, and anti-apoptotic defense pathways (Ortiz et al., 2003; Gupta et al., 2007; Xu et al., 2007; Lee et al., 2009; Gotts and Matthay, 2011; Liu et al., 2017).

## Conclusions and Future Perspectives

The imbalance of the redox system induced by the ECC is a field of active research, but many aspects of neonatal ECMO and its potential harm remain unsolved. Key points for both caregivers and researchers of the perinatal area addressing the burden of ECMO should include the following:

• Improve our knowledge of the mechanisms of oxidative stress during neonatal ECMO, both in pre-clinical and clinical settings, with a focus on the biochemical and cellular mechanism that could contribute to multi-organ damage.
• Define biomarkers for oxidative stress that could be a potential target for pharmacological approaches.
• Support technological research to improve ECMO circuit miniaturization and biocompatibility.
• Prevent the redox state unbalance during ECMO, through the promotion of rational use of blood products, the maintenance of well-defined O_2_ targets, the prevention of hemolysis and AKI, the timely resort to in-line renal replacement therapy, the simplification of ECMO circuit lines.

## Author Contributions

GC, GR, SG, SP, and FM conceived and designed the review. GC, GR, and SG wrote the first draft of the manuscript. All authors contributed to manuscript critical revision, read, and approved the submitted version.

## Conflict of Interest Statement

The authors declare that the research was conducted in the absence of any commercial or financial relationships that could be construed as a potential conflict of interest.

## Abbreviations

AKI: acute kidney injury
ARDS: acute respiratory distress syndrome
ATP: adenosine triphosphate
CAT: catalase
CPB: cardiopulmonary bypass
CRRT: Continuous Renal Replacement Therapies
Cys–S^-^: cysteine thiolate anion
Cys-SO_2_H: cysteine sulfinic acid
Cys-SO_3_H: cysteine sulfonic acid
Cys–SOH: cysteine sulfenic acid
d-ROMs: reactive oxygen metabolism and derivatives
ECC: extracorporeal circulation
ECMO: extracorporeal membrane oxygenation
ELSO: extracorporeal life support organization
Fe^2+^: ferrous ions
Fe^3+^: ferric ions
FO: fluid overload
GPx: glutathione peroxidases
Grx: glutaredoxin
GSH: glutathione
H_2_O_2_: hydrogen peroxide
hMSCs: hypoxia-preconditioned mesenchymal stem cells
IFNβ: interferon β
IL: interleukin
MAS: meconium aspiration syndrome
MDA: malondialdehyde
MOF: multi-organ failure
NAC: N-acetylcysteine
NADP^+^: nicotinamide adenine dinucleotide phosphate oxidated form
NADPH: nicotinamide adenine dinucleotide phosphate reduced form
NEC: necrotizing enterocolitis
NO: Nitric oxide
NOS: nitric oxide synthase
NOX: NADPH oxidase
O_2_: oxygen
O_2_^-^: superoxide anion
OH: hydroxyl radicals
ONO_2_^-^: peroxynitrite
PCs: platelet concentrates
PFHb: plasma free hemoglobin
PPH: persistent pulmonary hypertension
PRBC: packed red blood cells
PRx: peroxiredoxins
PUFA: polyunsaturated fatty acid
pVWF: plasmatic Von Willebrand factor
RBCs: red blood cells
RDS: respiratory distress syndrome
RNS: reactive nitrogen species
ROS: reactive oxygen species
Se: selenium
SIRS: systemic inflammatory response syndrome
SOD: superoxide dismutases
TAC: total antioxidant capacity
TF: tissue factor
TNFα: tumor necrosis factor α
TR: thioredoxin reductase
Trx: thioredoxin
VA-ECMO: venous-arterial ECMO
VV-ECMO: venous-venous ECMO
