# Supplementary material for: Oxidative Stress and Neonatal Respiratory Extracorporeal Membrane Oxygenation
Source: Front Physiol. 2018 Dec 4;9:1739. doi: 10.3389/fphys.2018.01739 (PMC6288438; doi:10.3389/fphys.2018.01739)
Supplement: Supplementary file 1 [file Table_1.docx]

|  |  |  |  |  |
| --- | --- | --- | --- | --- |
| ***Table 1. Antioxidants: main evidence from clinical and pre-clinical studies in the critically ill and/or exposed to an extracorporeal circuit.*** | | | | |
| **Antioxidant** | **Disease** | **Population** | **Outcome of the study** | ***Reference*** |
| Selenium (alone or + micronutrients) | CPB-cardiac surgery | Adults | Improvement of clinical outcome at post-operative day 1 but not confirmed in the entire observation period. | *Stoppe et al. 2013* |
|  | Critically ill | Adults | Significant reduction in mortality and mechanical ventilation days. Trend toward reduced infections. No overall effect on hospital length of stay. | *Manzanares et al. 2012* |
|  | Sepsis | Newborns | Significant reduction in the proportion of preterm infants having one or more episodes of sepsis. | *Darlow et al. 2003* |
| Vitamin C | Surgery | Adults | Decrease of incidence of postoperative atrial fibrillation. | *Carnes et al. 2001* |
|  | Hemodialysis | Adults | Reduction of levels of oxidative stress (evidenced by markers of lipid peroxidation) | *Eiselt et al. 2001* |
|  | Critically ill | Adults | Restoration of endothelial function and modulation of redox signaling | *Wilson et al. 2009* |
| Vitamin E | Hemodialysis | Adults | Decrease of d-ROMs and increase of TAC and SOD. | *Takouli et al. 2010* |
| NAC | Surgery | Adults | Decrease of the incidence of postoperative atrial fibrillation. | *Ozaydin et al. 2008* |
|  | NEC | Murine neonatal model | Improvement of anti-inflammatory (decreased TNF-alfa) and antioxidant (increased SOD activity) properties in the gut. | *Ozdemir et al. 2012* |
| Glutamine (alone or +arginine) | CPB-cardiac surgery | Adults | Increase in glutamine level and in GSH activity. | *Engel et al. 2009* |
|  | NEC | Murine neonatal model | Glutamine: increase of intestinal SOD and GPx levels and decrease in MDA levels.  Glutamine + arginine: increase of GPx activity and decrease of MDA level. | *Kul et al. 2009* |
| Melatonin | Sepsis | Newborns | Reduction of lipid peroxidation products (MDA) and improvement of clinical outcome | *Gitto et al. 2001* |
|  | Surgery | Newborns | Reduction of pro-inflammatory cytokines and NO levels | *Gitto et al. 2004* |
| Apocinine | Cerebral stroke induced by ischemia-reperfusion | Murine model | NADPH oxidase Inhibition, reduction of brain Infarction and improvement of neurologic functions | *Chen et al. 2009* |
| α-lipoic acid | Surgery + ECC | Adults | Decrease of IL-6 and IL-8 and decrease of C3 and C4 level | *Uyar et al. 2013* |
| Lactoferrin | Sepsis | Newborns | Decrease of late-onset sepsis risk ratio | *Pammi et al. 2017* |
| Zinc | Sepsis | Newborns | Reduction of the risk of antibiotic treatment failure | *Bhatnagar et al. 2012* |
| Edavarone | Sepsis | Murine neonatal model | Clinical improvement of septic animals (higher cardiac output and mean arterial pressure, lower heart rate, and longer survival time) | *Kato et al. 2009* |
| C3: complement 3; C4: complement 4; CPB: cardiopulmonary bypass; d-ROM: reactive oxygen metabolites and derivatives; ECC: extracorporeal circulation; GPx: glutathione peroxidase; GSH: glutathione; MDA: malondialdehyde; NAC: N-acetylcysteine; NEC: necrotizing enterocolitis; NO: nitric oxide; TAC: total antioxidant capacity; SOD: superoxide dismutase. | | | | |
